# On an RNA-Membrane Protogenome

**DOI:** 10.3390/life15050692

**Published:** 2025-04-24

**Authors:** Michael Yarus

**Affiliations:** Department of Molecular, Cellular and Developmental Biology, University of Colorado, Boulder, CO 80309, USA; yarus@colorado.edu; Tel.: +1-303-817-6018

**Keywords:** RNA world, RNA9, RNA10, DNA, lipid, phospholipid, fatty acid, bilayer, raft, genome, division, fusion, evolution, anthology, Standard Genetic Code

## Abstract

Efficient evolution exists before DNA, else the DNA genome itself could not evolve. Current data suggest RNA-membranes for this role. Selected RNAs bind well to phospholipid bilayers; randomized sequences do not. No repeated sequences are evident in selected binding RNAs. This implies small and varied membrane-affinity motifs. Such binding sequences are partially defined. Phospholipid-bound RNAs require divalents like Mg^2+^ and/or Ca^2+^, preferring more ordered bilayers: gel, ripple, or rafted membranes, in that order. RNAs also bind and stabilize bent or sharply deformed bilayers. RNA binding without divalents extends to negatively charged membranes formed from simpler anionic phospholipids and to plausibly prebiotic fatty acid bilayers. RNA-membranes frequently retain RNA solution functions: base pairing, passive transport of tryptophan, specific affinity for arginine side chains, and ribozymic ligase catalysis. Membrane-bound RNAs with several biochemical functions, linked by specific base-pairing, are readily constructed. Given these data, genetic roles seem feasible. RNA activities often require few nucleotides, easily joined in a small RNA. Base-paired groups of such RNAs can also be purposeful, joining related functions. Complex functions can therefore require only replication of short RNAs. RNA-membranes potentially segregate accurately during cell division and quickly evolve through new base pairings. Accordingly, ancient RNA-membranes could act as a protogenome, supporting encoded RNA expression, inheritance, and evolution before the DNA genome: for example, supporting organized biochemistry, coded translation, and a Standard Genetic Code.

## 1. Introduction

**Figure 1:**
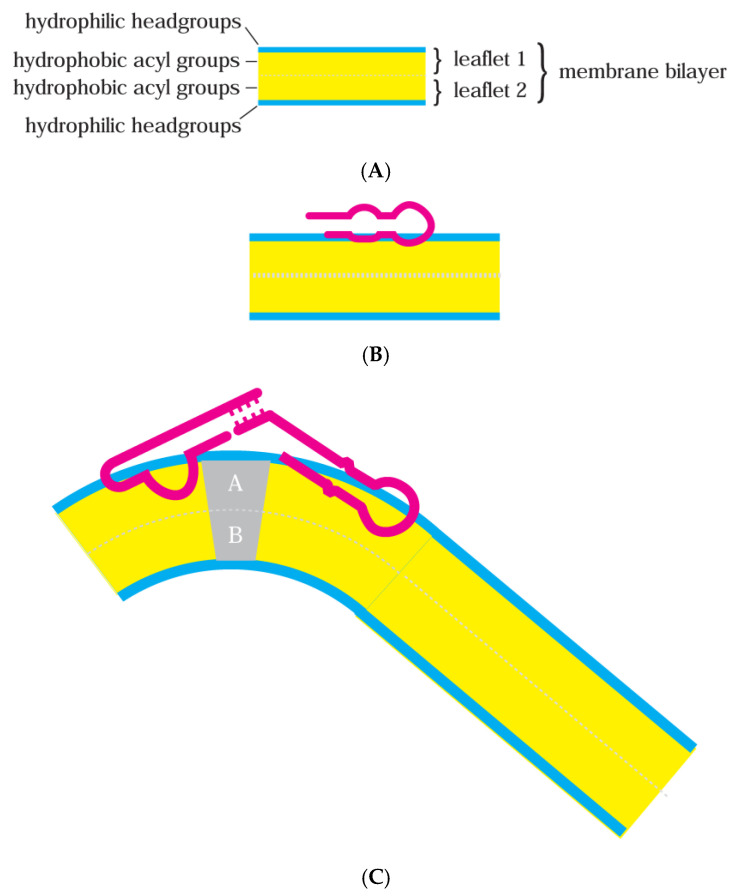
(**A**) Simplified cross-section for a bilayer lipid membrane. The membranes discussed herein, for example, can be composed of palmitic acid and a saturated 16-carbon fatty acid whose headgroup (blue) is a negatively charged carboxyl and whose acyl group (yellow) is a 14-carbon single-bonded chain of –CH_2_, terminated by –CH_3_. Modern phospholipid membranes contain more complex phosphatidyl cholines, with headgroups (blue) of positively charged choline linked to negatively charged phosphate linked to glycerol; they are therefore zwitterionic, overall electrostatically neutral with large dipole moments. Hydrophobic acyl groups esterified to glycerol in modern phospholipids (yellow) can be, e.g., palmitic acid plus oleic acid, an 18-carbon fatty acid kinked by one double bond at carbon 9. (**B**) A bilayer with bound RNA. RNA is simplified to a broad red backbone. Bound RNA is confined interfacially near the lipid headgroup in the initially contacted leaflet, as shown. Thus, RNA still contacts the solution phase, consistent with limited changes in both RNA and the membrane during membrane-RNA formation. (**C**) A distorted membrane-RNA. A bilayer with bound RNAs, stabilizing a bent membrane conformation. Leaflets A and B can contain lipids in the gray region with particular shapes, favoring bent bilayers. Though multiple different active RNAs are shown, membranes can also be deformed by multiple single RNAs.

This text has three parts: First, reviewing experimental interaction of lipid bilayer membranes (Figure 1A) and RNA (Figure 1B). Then, the (Discussion), suggesting that known RNA-membrane biochemical activities suffice for heritable effects. Lastly, suggesting a role for RNA-membranes during early code evolution (Discussion). First, the review.

### 1.1. Membrane-Binding RNA Selection

Selection of membrane-bound RNAs (Figure 1B) from random sequence molecules clarifies RNA-membrane evolution. Free RNAs are smaller than liposomes, so readily resolved through size-exclusion chromatography [1] from first-eluted liposomes. Using liposomes with fluid phosphatidylcholine–cholesterol membranes, RNAs with 50 internal randomized nucleotides were selected for migration with liposomes. Initial randomized RNAs did not detectably bind. But after 11 cycles of selection, 70% of individual sequences stably re-bound to liposome exteriors, requiring 20 mM Mg^2+^ and 10 mM Ca^2+^. Four out of the eight tested RNAs disrupted bilayers, releasing ^22^Na^+^ from liposomes. Thus, four out of the eight selected RNAs bound without disturbing membrane permeability. Selected RNAs also acted on intact biological membranes, making patch-clamped HEK293 cells conductive. Smaller RNAs are functional; 53- and 44-ribonucleotide truncates were membrane-active.

Selected RNAs foreshadowed later isolates: no repeated sequences were evident [1] among the selected sequences, though tracts of repeated purines and pyrimidines were frequent. Thus, though membrane-binding sequences are not evident among randomized RNAs, they are readily selected. Secondly, 5 mM choline (but not homologous ethanolamine) prevents binding. Thus, RNA affinity for the polar headgroup phosphatidylcholine likely has a major role in RNA binding. Thirdly, membrane-bound RNAs usually stay in the leaflet first encountered (Figure 1B); they remain outside exposed vesicles [1], exposed to exterior agents like ions and macromolecules like enzymes.

The conclusion that membrane interactions are mediated by small, varied sequences is supported by multiple efforts tracing bilayer interactions to repeated RNA motifs. These range from the above repetitive purine and pyrimidine tracts [1], to repeated AG [2], G quartets [2], to UUGU, UCCC, CUCC, CCCU [3,4], to CCCU and GGAG [5], to UUUCU, UUCAC, UUGCAC, UUUUCC, and UCUCU [6]. The sequence CAAUUCCAG is protected from digestion after RNA-membrane association [1]. However, membrane-bound RNAs exist that contain none of these [1].

But membrane binding can also be chemically simple, conferred by a small hydrophobic group. Biological human tRNA^Sec^ binds to HeLa lipid liposomes [7], but a transcript of the same sequence does not. A five-carbon isopentenyl anticodon loop modification of human tRNA^Sec^ probably inserts into liposome membranes, conferring a mean residence of ≈5 min at 24°. Study of tRNA affinity for membranes [8] may constitute measuring a sum of RNA and hydrophobic modification effects and may therefore be challenging to interpret.

### 1.2. Multiple Lipid Forms

Biological compartments, delimited by bilayer membranes, have a hydrophilic exterior contacting the aqueous environment on both sides and a hydrophobic membrane interior that obstructs water and other polar molecules (Figure 1A). Modern cell outer membranes and the borders of cell organelles contain phospholipid layers that are not necessarily uniform and planar but instead intermix multiple lipid conformations [9,10].

Fluid—least ordered of the bilayer membrane forms, allowing lipid molecules to diffuse freely and change in conformation, with acyl chains isomerizing from extended to kinked.

Liquid-ordered—an intermediate membrane with freely-diffusing lipids having extended orderly acyl groups within a bilayer; these are irregularly intermixed with planar, more fluid regions.

Ripple—some fluid lipid bilayers, when cooled, congeal into corrugated intermediate regions with some ordered and some less ordered lipid forms alternating in regular ridges [11].

Rafted—areas of lipid that intermix sphingolipid, cholesterol, and common phospholipids in a more ordered complex, occurring as ordered islands within a more fluid bilayer [12]. Rafts concentrate cellular molecules to perform essential biochemical functions.

Gelled—near-solid condensed immobile lipids below their major melting transition, constituting a maximally ordered bilayer.

The lipid forms above are listed, top to bottom, with increasing order [13]. This distinction is useful because RNA prefers binding to more ordered phospholipid layers [14]. Typical bench experiments controllably decrease membrane order by increasing temperature, using unsaturated rather than saturated fatty acids, employing shorter acyl groups that support weaker interior hydrophobic bonds, and reducing Ca^2+^ or increasing Na+ [15].

### 1.3. Molecular Dynamics on RNA-Membranes

Phospholipid binding is clarified by recent molecular dynamics [16,17] studies of RNA and lipid bilayers. Among ribooligonucleotides, only (pG)_4_ stably binds to gel phospholipid membranes, whereas tetra-A, -C, and -U reside only transiently on gel membrane surfaces. Affinity relies on surface electrostatic and Van der Waals contacts between G bases and the phosphatidylcholine headgroups of the RNA [17]. Moreover, when the bilayer is fluid instead of gelled, binding of (pG)_4_ weakens and becomes transient. These calculations therefore rationalize the inhibition of RNA binding by a lipid headgroup analogue [1], the presence of runs of G among membrane RNAs [1,2], and the preference of RNAs for structured lipids [14]. The superiority of G base–lipid hydrogen bonding can be confirmed even for nucleoside–membrane interactions [16], and it appears that A bases may uniquely insert into lipid surfaces, due to the unstable hydration of A [16]. Though hydrophobic interactions of bases with lipid acyl chains can be envisioned [18], molecular dynamics presently allows for the interpretation of RNA-membrane interactions solely in terms of structural and chemical matching of polar headgroups, ions, and RNA bases (especially G), though this leaves the above variety of sequences associated with RNA-membrane binding to be explained.

### 1.4. Fluid Membrane Affinity

**Figure 2:**
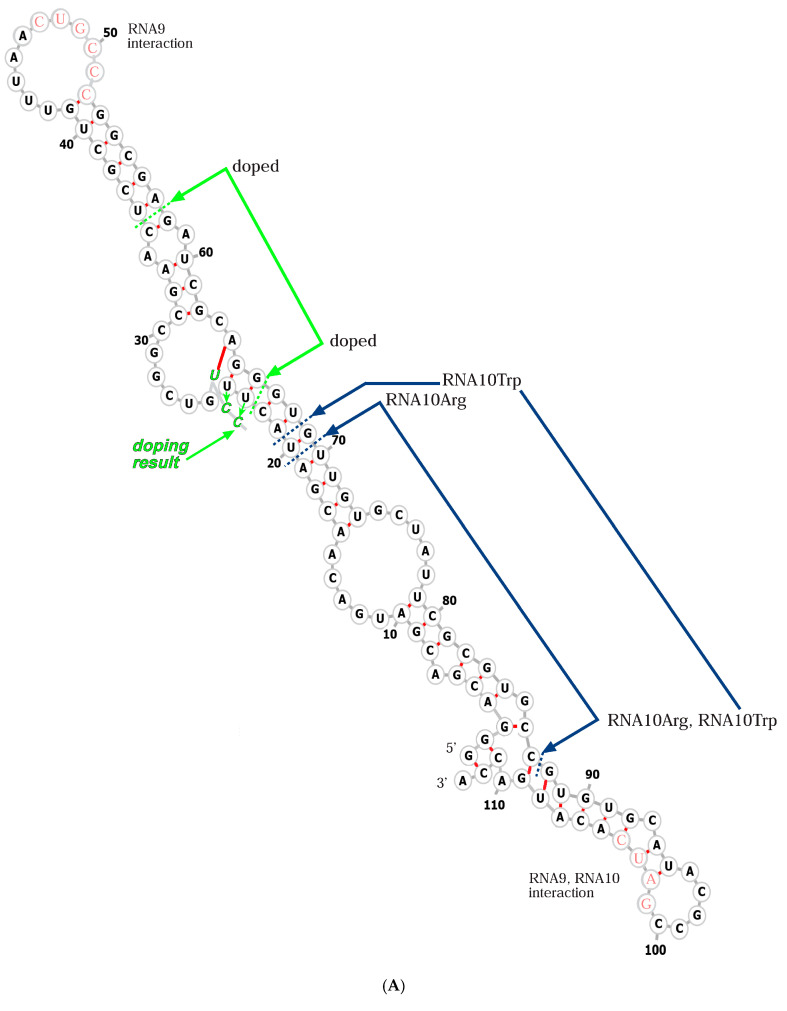

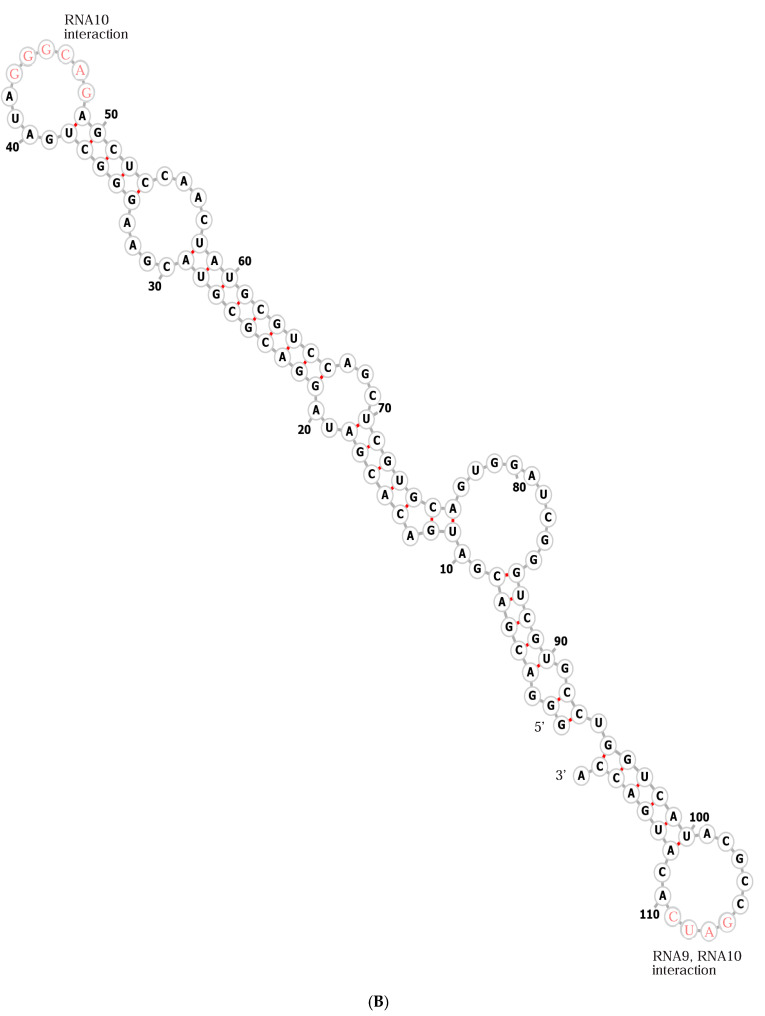
(**A**) RNA10 sequence and activity. A fold for the RNA10 113-mer [19] is shown, derived from a minimum free energy condition [23], with added structures from trRosettaRNA [24] and reconciliation with data on inter-nucleotide flexibility [19]. The structure is marked to show RNA9:RNA10 interaction sequences in pink lettering [19] and with dashed lines to identify regions replaced (blue marked arrows) by a tryptophan site in RNA10Trp [21], the region replaced by an arginine site in RNA10Arg [22], and the region of RNA10Arg doped and reselected (green arrows) for improved membrane and arginine affinity, beginning with 85% of the original nucleotide and 5% of each alternative. The improved sequence derived after doped reselection [22] is also shown with green lettering. Both 5′ and 3′ termini were unselected, fixed sequences favoring sequence reproduction. (**B**) The RNA9 sequence. An estimated minimal free energy fold [23] is shown.

Stable phospholipid membrane binding selections in RNA solutions with reduced divalents (5 mM Mg^2+^ and 2 mM Ca^2+^) do not recover single RNAs [19]. Instead, individual RNAs must interact to bind efficiently and alter fluid phospholipid membrane permeability (Figure 1C). One efficient pair is RNA9:RNA10 (Figure 2A,B), which binds optimally if supplied in a 2:1 molar ratio, though weak fluid membrane affinity is detectable for RNA9 alone under these conditions. Native gel electrophoresis suggests that an RNA9:RNA10 kissing loop [20] complex oligomerizes further when it binds fluid phosphatidylcholine membranes stably. RNA9:RNA10 oligomers also disrupt black membranes and release interior liposome GT^32^P; they can therefore probably stabilize transient membrane pores. Selection of only RNA multimers emphasizes simple RNA combinations; selected partners must be frequent enough in randomized RNAs to reproducibly oligomerize during selection.

RNA9:RNA10 membrane oligomers are confirmed using tapping-mode atomic force microscopy, using flattened phosphatidylcholine vesicles supported on hydrophobic mica [13]. RNA10 alone on mica appears as distributed individual monomers. RNA9 on mica is in varied chains, but if its RNA9:9 kissing loop interaction with itself (Figure 2B) is disrupted by mutation of two nucleotides, it appears as monomer RNAs. In contrast, mixed RNA9:RNA10 on mica is very heterogeneous, with 29 weight percent as longer complexes, including chains. Most interestingly, in the lipid vesicle layer, mixed RNA9 and RNA10 oligomerize further, as thick bands selectively concentrate at lipid patch edges. Apparently, such RNAs prefer unusual lipid conformations in sharply bent membranes at flattened liposome peripheries.

This is further emphasized by fluorescence microscopy of labeled RNA9:RNA10 bound to distinctly fluorescent membranes (Figure 2 of [13]). RNA appears throughout membranes, tracking membrane fluorescence accurately, but also concentrates at bends. For example, it is particularly intense at the constriction between joining or separating vesicles. This predicts structural effects: affinity for curved bilayers enables RNAs to favor not only transient pores but also membrane fusion or division (Figure 1C).

### 1.5. RNA9:RNA10

RNA sequences (Figure 2) rationalize membrane functions. RNA10 (Figure 2A) is an extended 113-mer. Widely separated small sequences apparently account for oligomerization on membranes; for example, RNA10 pairs to RNA9 via the sequence CUGCCC at nt47 (Figure 2A), which is chemically protected when RNA10 pairs with RNA9. Also, a deoxyoligonucleotide of similar sequence prevents RNA10:RNA9 interaction [19]. The antiparallel RNA9 complement GGGCAG (Figure 2B) is also protected by RNA10:RNA9 pairing. RNA9 118-mer forms a stable dimer with itself [19] at the self-complementary tetramer GAUC at nt106 (also present near the constant 3′ terminal sequence of RNA10). An overlapping deoxoligonucleotide prevents RNA9 dimerization, as does mutation of RNA9 GAUC nucleotides [19].

**Figure 3:**
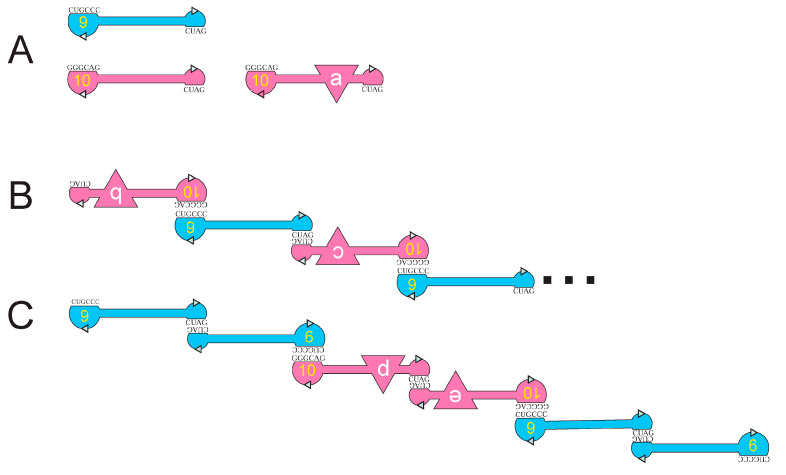
(**A**) Simplified graphics for RNA9 and RNA10 structures. RNA9 is shown in blue, RNA10 and functionalized RNA10 [21,22] are shown in red. Yellow numerals in larger loops are RNA names. Figure boundaries represent RNA backbones, and green arrowheads show intrinsic oligoribonucleotide polarities. Small black letters beside flattened loops are complementary RNA sequences. Triangular intercalations are internal functional RNA domains like those already known [21,22], with functions a, b, c, d, and e. RNAs bearing such domains are called RNA a, RNA b… in the text. (**B**) An RNA9:RNA10 complex yielding chains of arbitrary length [13]. The structure shown can be extended by an arbitrary number of RNA10:RNA9 dimers, symbolized by dots on the right. (**C**) An RNA9:RNA10 complex with observed stoichiometry—2 RNA9: 1 RNA10 [13].

These observations are combined in Figure 3, which radically simplifies RNA9 (Figure 2B) and RNA10 (Figure 2A) sequences as outlines in order to clearly depict their association, implicated in membrane interaction [19] and strongly constrained by RNA polarities and sequences. Figure 3’s outlines represent RNA backbones, which have polarities indicated by 3′-directed green arrowheads in boundary lines.

In Figure 3A, individual RNA outlines are presented, each named with a yellow number within its larger loop; RNA9 is blue and RNA10 is red. As mentioned above, complementary interacting sequences are presented beside larger (leftward) and smaller (rightward) kissing loops. RNA10 is shown in two forms, with the leftward red form being the initially selected RNA10 (Figure 3A). The rightward red outline is an experimentally composed RNA10 with an inserted triangular functional domain replacing some initial RNA10 sequences (Figure 3A), as in a passive RNA-membrane transporter that binds tryptophan [21] or adds specific arginine affinity to a bilayer [22].

In Figure 3B,C, membrane-active RNA9:RNA10 complexes are shown, paired via loop sequences highlighted in Figure 2A,B. Native gel electrophoresis [19] and atomic force microscopy [13] consistently suggest RNA9 alone forms a stable dimer with itself. RNA10 alone is a monomer, probably because its GAUC is engaged in secondary structure (Figure 2A). When RNAs are mixed, varied, larger aggregates are formed as chains and more complex shapes [13]. Size-exclusion chromatography chooses stable binding because early eluted liposome-bound RNAs must survive in the absence of unbound RNA. RNAs bind optimally when a stoichiometry of 2 RNA9:1 RNA10 is supplied; recovered membrane-bound RNAs have the same 2 RNA9: 1 RNA10 stoichiometry [19].

Figure 3B shows RNAs that can form chains of any length by repeating an RNA10:RNA9 unit, as suggested by the triple dots in the Figure on the right. Thus, Figure 3B explains the observed long chains [13], which would have 1:1 stoichiometry.

Figure 3C shows a complex of stoichiometry 2 RNA9:1 RNA10, consistent with this property in liposome-bound RNAs. Figure 3C uses the observed stable RNA9 dimer at both ends, paired via hexanucleotide kissing loops to a central RNA10 dimer. Both dimers are paired via GAUC self-complementarities. Figure 3C predicts a stable RNA9:RNA10 dimer in the presence of the GAUC loop DNA oligo competitor, as observed [19]. Figure 3C’s structure plausibly dominates bound RNA stoichiometry; in fluid phospholipid membranes, RNA9 supplies membrane affinity, while unaltered RNA10 is not stable on liposomes [19]. Figure 3C is the RNA complex with the greatest RNA9:RNA10 ratio; it might well bind most quickly and be the major RNA retained on resolved fluid liposomes.

RNA9:RNA10 complexes on fluid membranes [13] apparently retain base pairing like that in solution [19]; we will return to this in the Discussion.

### 1.6. Bound RNAs Also Retain Complex Solution Functions

RNA-membranes also retain RNA functions requiring preservation of larger solution structures. An initial example was the passive membrane transport of tryptophan by an RNA [21]. An internal section of RNA10, which had given no biochemical indication of a membrane role [19], was replaced (Figure 2A) with a Trp binding site [25]. This modified RNA10Trp (Figure 2A) still bound fluid liposome membranes, aided (Figure 2B) by RNA9 [19]. Tryptophan binding was also intact, though with a higher K_D_. Nevertheless, the amino acid site was still Trp-specific, both complexed in solution and when membrane-bound. Liposome-bound RNA9:RNA10Trp admitted the amino acid to liposomes at an enhanced rate, measured both isotopically and by increasing Trp FRET to an internal liposome chromophore. Liposome fusion was ruled out by controls, and calculated Trp transport was comparable to passive protein transporters using similar mechanisms.

Similarly, RNA10Arg, built using a similar replacement of an interior RNA10 sequence with an RNA site for Arg (Figure 2A), retained arginine specificity, with it being unresponsive to eight other amino acids [22]. Interestingly, a distinct internal tract in RNA10Arg (Figure 2A) was resynthesized (doped) with 5% of the other three nucleotides at all positions and reselected, yielding increased membrane and Arg affinities. This included a UUG to CCCG alteration (Figure 2B) that enabled reselected RNA10Arg to bind rafted and rippled phospholipid bilayers without RNA9 assistance.

Moderate conformational change in RNA-membrane formation is readily detected in ribozyme activities. Hammerhead self-cleaving ribozyme can be assembled from fragments that are inactive and then activated by binding to zwitterionic phospholipid liposomes [26]. The R3C ligase ribozyme [27] is active on membranes [2]. If ribozyme ligation is inhibited by the addition of a terminal membrane-affinity sequence, ligase recovers its activity when the inhibitory sequence is sequestered by membrane binding.

### 1.7. Bound RNAs Shape Bilayers

Bound RNAs take up space in the membrane leaflet first contacted (Figure 1B). Bound RNAs enlarge their contacted leaflet; thus, they tend to bend contacted bilayers away from themselves.

Moreover, membrane lipids have intrinsic shapes that can reinforce RNA effects. Membrane lipids can have small headgroups (conical lipids) or small hydrophobic acyl groups (reverse conical lipids) and so induce their bilayer leaflet to bend [28]. Alternatively, headgroups and acyl groups balance (cylindrical lipids), favoring a flat membrane. Figure 1C depicts bending, favored by two base-paired, bound RNAs and aided by specific lipids in membrane regions A and B. For example, in Figure 1C, large lipid headgroups in A and large acyl groups in B (Figure 1C) could collaborate with paired membrane-bound RNAs to curve the bilayer.

### 1.8. Rafts Uniquely Distinguish RNA Sequences

RNA-membrane interaction strongly responds to membrane lipid structure. Using phosphatidylcholine liposome membranes with different acyl groups, at different temperatures, ± cholesterol to favor lipid order, chemically similar membranes in differing structural states were compared for RNA affinity [14].

Specifically, RNA10 [19], which stably adhered to fluid phosphatidylcholine membranes only when associated with RNA9 [19], now binds unassisted to more ordered bilayers. Both rippled (DMPC @ 18–23°) and rafted (6 DOPC: 3 sphingomyelin: 1 CHOL @ 23°) membranes bind RNA10 but release it when the temperature is raised, making these bilayers fluid. Because the temperatures used are well below RNA T_m_, these are probably effects on membrane structures. Interestingly, all tested RNAs bind ≈ equivalently to rippled bilayers, but rafted bilayers make strong structural distinctions. For example, rafts bind forms of RNA10 well but bind randomized-sequence RNAs very poorly. Rafted bilayers uniquely show RNA structure-specific affinities, noted in the Discussion.

### 1.9. Singly Charged Lipid Headgroups, Including Fatty Acids, Also Form RNA-Membranes

The above phenomena occur on complex neutral zwitterionic phospholipid membranes, prevalent in modern cells. However, interaction with RNA extends to simpler lipid bilayers, more plausible during early cellular life.

Cationic dioleyl trimethyl ammonium propane bilayers on quartz surfaces stably bind RNA9 with RNA10 [19] under simple conditions, with only buffer and Mg^2+^ present [29]. This interaction is presumably principally mediated by electrostatic complementarity between negative RNAs and a positive membrane lipid headgroup. In contrast with moderate conformational effects on zwitterionic membranes, such large-scale electrostatic complementarity to a positive headgroup strongly alters both tRNAs and an 861-nucleotide transcript [30], denaturing them.

Anionic, negatively charged phosphatidyl glycerol membranes bind RNA10 in buffer and NaCl [31]. The membrane somewhat destabilizes the RNA to melt as the temperature increases, and bound RNA10 simultaneously stabilizes the membrane lipid structure and increases its cooperativity during bilayer melting. Thus, uniform negative membrane surface charge is not a bar to RNA interaction, and (unlike zwitterionic phosphatidylcholine bilayers), divalent ions are not required for RNA interactions that somewhat change both RNA and lipid reactants.

Another kind of anionic extension exists for saturated 16-carbon palmitic acid, a possibly primitive [32] bilayer-forming fatty acid. Using RNA binding to liposome-coated magnetic beads (in buffer, Mg^2+^ and Ca^2+^ at 24°), such fatty acid membranes bind randomized RNA sequences significantly better than control fluid membranes; in fact, only ≈four-fold less well than rippled phosphatidylcholines [2]. Affinity for randomized sequences clearly suggests common, varied RNAs with affinity for palmitate membranes.

Accordingly, fatty acid-RNA interactions exist in candidate primordial membranes. Such bilayers are more fragile but more permeable, which may serve the metabolic needs of early cells [33], for example, by improving access to ribose [34]. Fatty acid bilayers are also sensitive to Mg^2+^, often required for RNA activities. But such sensitivity is relieved by mild chelation of the divalent [35], in membranes composed of varied lipid types [36], or in special environments [37]. Fatty acid bilayers therefore exemplify primordial cell boundaries in the Discussion below.

## 2. Discussion

### 2.1. A Unified Binding Mechanism

Much about RNA-membrane interaction appears to be an effect of fitting RNA to a partially structured binding site. That is, the cost of rearranging lipids contacting bound RNA appears highly significant. For this reason, RNAs bind bilayers best (and independent of RNA structure) if bilayers are gelled strongly but somewhat less if bilayers are rippled and bind less well but discriminate RNA structures if rafted. Weakest binding occurs to fluid bilayers, where lipid rearrangements are most extensive and costly. Thus, the entropic cost of fixing otherwise somewhat free lipids and the enthalpic penalties for forcing lipids and RNAs into a mutually less favored bound environment dominate RNA affinity. It is presently possible that most such accommodations will involve RNA backbones and bases adapting to polar lipid headgroups, and perhaps their ions [16,17], but highly varied known bilayer-binding sequences offer many openings for novel soft-matter biochemistry and biophysics.

Lipid rearrangements also account for the accumulation of RNAs on sharply bent fluid membranes, like the junctions of fusing or dissociating vesicles, similarly explain RNA9:RNA10 oligomer preference for the edges of flattened vesicles where membranes turn downward sharply. Geometric deformation of lipids mimics the loss of lipid freedom in more ordered membranes [13]. RNA prefers bilayers less free to change. However, rafts are a special case [14]. Lipid adjustment with differing RNAs is discriminating: rafted phospholipids apparently bind RNA structures, demanding the most favorable rearrangements.

**Figure 4:**
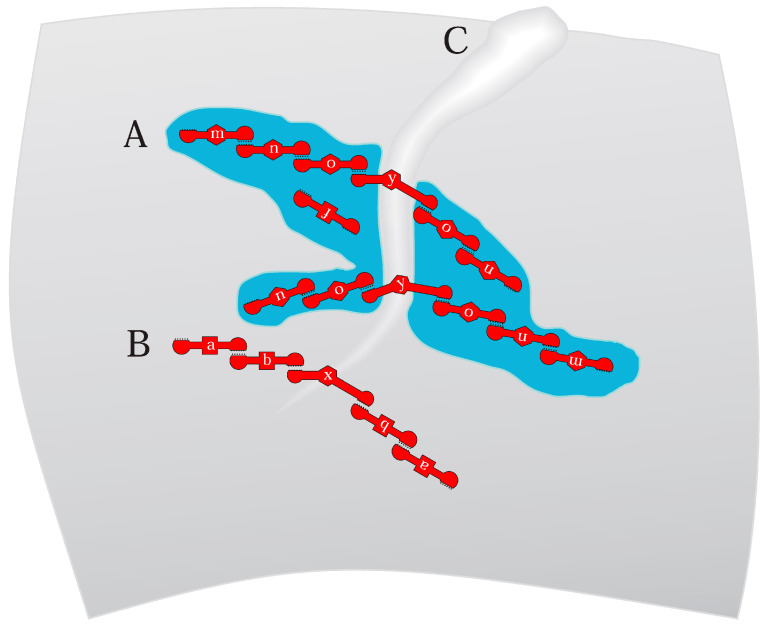
(**A**) The RNA-membrane protogenome hypothesis. The curved gray background is the inner side of an RNA cell [38] boundary. Blue regions are rafted bilayer domains. Red objects are simplified RNA molecules using a notation resembling Figure 3; functionalized RNA10-like RNAs with terminal loops with complementary sequences and internal functional domains a, b, and f or m, n, and o as in RNA10Trp [21] and RNA10Arg [22] structures (Figure 2). Functional domains x and y resemble Figure 1C, binding and stabilizing [13] membrane deformations. (**B**) Protogenomic activity but with RNA groups bound to and segregating on the inner surface of a simpler, unrafted, perhaps fatty acid-like membrane [2]. (**C**) An inward-growing depression in the cell-bounding bilayer, ultimately capable of dividing the cell and membrane. RNAs may have initiated a division furrow or joined a nascent furrow, reinforcing it.

Lipids are observably changed around RNA binding sites. RNA10 [19] alters the temperature profile of bilayer melting [14]. In fluorescence microscopy, RNA10 [19] completely coats large rafted areas on vesicles [14], precisely tracking raft edges. Such coating is not definitive, but suggests that altered RNA-bound lipids may aid the fusion of smaller RNA-rafts to generate larger ones, an important consideration for joint RNA functions (Figure 4).

There is another way to view lipid preference. RNA affinity for constrained membranes implies that RNA binding will enhance unusual lipid arrangements. Accordingly, membrane RNAs can facilitate particular biological events (Figure 1C): pore formation, invagination/endocytosis, vesiculation/exocytosis, and tubulation. Symmetrical vesiculation resembles cell division; thus, RNAs potentially induce or guide membrane division [38].

### 2.2. Rafts Organize RNAs

In Figure 4A, interior blue leaflet regions are rafted, distinct from the major gray exterior membrane surrounding them. Smaller red structures are varied functional RNAs (with functions arbitrarily named a, b, m, n, and o) and also have small sequences required for rafted membrane affinity. Raft and functional sequences in one RNA are plausible (Figure 2 and Figure 3); membrane affinity requires few mutations in a free RNA [22]. Sequence-dependent raft affinities suggest that inherited rafted RNAs, co-existing with other cell RNAs lacking membrane affinity, are inherited less precisely.

### 2.3. Facilitating Replication

RNA catalysis is long known to include chemistry for inter-ribonucleotide bond formation [39]. But much creative work has been required to evolve a 182-ribonucleotide replicase that can accurately replicate a 34-ribonucleotide hammerhead ribozyme [40]; accurate replication of longer replicase itself, by itself, is still not possible. Perhaps a second medium joining smaller catalytic RNA fragments holds the solution. While not comparable in detail, distributed synthesis of longer RNAs within mineral galleries of montmorillonite clay [41] comes to mind.

However, RNAs of a few ribonucleotides in length still exhibit complex functions. A ribozyme consisting of tetramer and pentamer RNAs synthesizes tetramer aminoacyl-RNA, later readily converted to peptidyl–tetramer RNA [42]. These small RNAs can be combined with an activating ribozyme [43] to both activate and transfer an amino acid, synthesizing aa-RNA [44] and emulating both activities of modern protein aminoacyl RNA synthetases. The short pentamer ribozyme can be active when inserted into longer ribosomal RNA sequences [45]. Thus, joined active sequences assemble multi-functional, small RNAs.

Multi-functional RNAs facilitate RNA replication because fewer internucleotide bonds are inevitably more easily replicated. This is especially important in a primordial time when replication is inefficient. One can evolve complete inheritance statistically by making numerous copies of active RNAs and randomly dividing them. But before efficient replication, more accurate segregation (Figure 4) greatly decreases the need for profuse replication.

Accordingly, one readily envisions (Figure 4) short RNAs that specifically bind bilayers, contain varied functional domains, and further, pair to build extensive structures [21,22]. Figure 4 relies on modular RNAs that express complex functions by combining multifunctional, more easily replicated subsequences. Such RNAs must also maintain functional sequences that enable their reproduction, as experimental RNA9 and RNA10 derivatives must also do (Figure 2).

### 2.4. Coordination with Cell Division

Figure 4 RNAs x and y strongly prefer altered lipid conformations, resembling repeatedly observed RNAs (Figure 1C) that promote membrane curvature. A sufficient number of such RNAs can initiate a division furrow that expands to transect the cell (Figure 4C). Such events can also be aided by conical or reverse conical lipids that help a furrow form and extend (Figure 1C). In this RNA-initiated case, division is RNA-determined, as shown by RNA pairing in Figure 1C. Such a mechanism could manifest a true cell cycle, dependent on cyclic availability of RNAs x and y or suitable lipids (Figure 1C). With regard to timing, note that membrane residence can decrease RNA stability [46], suggesting an encoded means for time-limiting a series of protogenomic actions.

Alternatively, a division furrow initiated by other cell events will collect RNAs [13]. RNAs joining a forming furrow suggest dual RNA roles: as free structures and catalysts, and then [47,48], after division is initiated, as units of inheritance. Base pairing makes RNA segregation during division a matter of efficient biophysical chemistry rather than random assortment. Accordingly, RNA-membranes emulate the later DNA genome, while requiring only documented RNA and lipid activities.

Figure 4’s RNAs are poised for accurate division. RNA y necessarily ties its symmetrical paired groups across the nascent division furrow (Figure 4A). Stressed, unusually configured furrow lipids will not be rafted; instead, the furrow can be bordered on both sides by rafts. RNAs paired to RNA y bind flanking rafts, as observed for RNA10 [22]. Base-paired groups span the furrow due to symmetrical, outward-directed pairing by RNA y. RNA y-bound groups will therefore be divided as the furrow grows inward. Multiple RNA y groups (Figure 4) help ensure that the y function itself is inherited.

### 2.5. Coordination of Expression

RNAs add to rafted areas, guided by complementary base-pairing to an existing raft RNA. This means that Figure 4’s rafted RNAs can be related groups, linked by one or more small base-pairing sequences. Such linkage is not theoretical; such paired elements exist in the specific paired loops (Figure 2 and Figure 3) of RNA9:RNA10 [13,19]. Rafted groups, for example, might be agents in an RNA-mediated pathway (e.g., RNAs m, n, and o).

### 2.6. Reproducing RNA Groups

RNA groups are not replicated in the usual sense, but instead repeatedly assembled by ordered base pairing. Such mechanisms are accurate, but not completely reliable; Figure 4 depicts two potential errors. Firstly, RNA f (Figure 4A) binds rafts as does doped, reselected RNA10Arg [22], using independent raft affinity. Figure 4’s RNA f segregates imprecisely but can readily mutate to be inherited in a group [22]. In addition, because membrane residence makes minor changes in RNA activities, evolution is facilitated because RNA f can develop a useful function while free, later joining a group that transmits it.

Protogenome imprecision would also include missing RNAs. Note that the rafted RNA with the m function, a terminal grouped RNA, can be lost or absent, as it is in the large rafted groups shown in Figure 4A. However, loss can be opposed statistically by a few RNA m groups, paralleling RNA y above.

### 2.7. Rapid Evolution

Figure 4A,B protogenomes can rapidly adopt novel functions. Groups easily add new capabilities via new base-paired individual RNAs. For such an addition, rafted and unrafted groups share the advantage of reduced dimensionality. As more rapid RNA cross-linking is observed on membranes suggests [2], finding partners is easier on two-dimensional surfaces than in three-dimensional solutions. In addition, protogenome assembly would benefit from more effective RNA structure formation due to encapsulation itself [49], which also stimulates evolution by increasing variance among RNA phenotypes [50]. Figure 4’s RNA-membrane protogenome is intrinsically highly evolvable.

### 2.8. Fatty Acid RNA-Membranes

**Figure 5:**
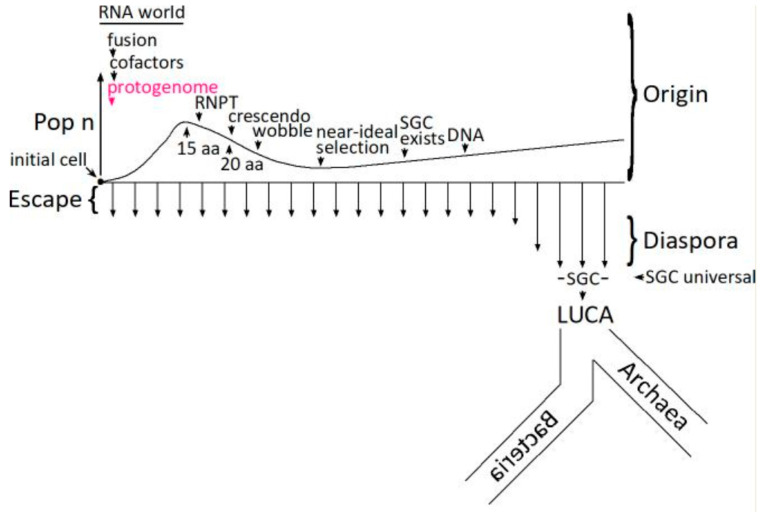
The protogenome in the evolution of the Standard Genetic Code. RNA-membranes facilitate the early evolution of the code. Terms and abbreviations in the Figure are defined by parenthetical entries in the Discussion section titled Relevance to Coding.

It is not clear whether all Figure 4 functions exist in simpler bilayers. Are there varied lipid structures, paralleling phospholipids, within fatty acid membranes? Fatty acids with varied headgroups and acyl chains suggest that inhomogeneous fatty-acid bilayers could exist (Figure 1C), but such possibilities must be experimentally explored. Perhaps chemically distinct Archaean ether-linked isoprenoid lipids [51] would be more appropriate (Figure 5). In any case, Figure 4B assumes RNAs on non-rafted surfaces, assuming only general membrane affinity, as already frequently observed among randomized RNAs [2]. Functional RNAs a and b pair with an RNA with membrane-distorting function x, as in Figure 1C. In Figure 4B, accurate RNA division rests on symmetrical outward-directed pairing (Figure 3) by furrow-binding RNA x. More specific primordial lipid RNA-membrane discussion awaits more data.

### 2.9. Biology as Anthology

Anthology implies combining characters that have evolved separately to quickly assemble a fitter genotype. An RNA protogenome rationalizes assembly of favored qualities before DNA, via RNA base pairing and shared bilayer affinity. In this context, cell fusions greatly accelerate protogenomic evolution [48], potentially anthologizing protogenomic advances from an entire community of related microbes [47,48] by joining paired RNAs and/or groups in the fused protogenome (Figure 4).

### 2.10. Relevance to Coding

Beneficial anthology extends to the Standard Genetic Code (Figure 5). Figure 4’s RNA groups seem particularly appropriate for the assembly of short RNA sequences for aminoacyl-RNA synthesis [45] and for direct RNA templating of primordial peptides [52,53].

Given improbable primitive chemistry and biochemistry, coding necessarily begins with a single initial cell (Figure 5). The number of cells in an initial coding population (Pop n) therefore begins at one. The code can be developed using only descendants of an initial improbable encoding cell [48], fusing to assemble a complete SGC in an increased number of more broadly coding cells (Pop n). However, cell fusion with independently arising codes is also efficient [48], reducing cell number (Pop n) and yielding complete codes originating almost completely from fusion [54]. Such fusions become especially significant during the RiboNucleoPeptide Transition (RNPT) [55], making coding more complete [56], and focusing on a consensus Standard Genetic Code [57]. The RNPT encodes newly biosynthesized amino acids [55,58], made with the aid of the earliest RNA catalysts (15 aa encoded to 20 aa), 5′-5′ ribonucleotide cofactors [59,60]. In an RNA world [38,61,62], therefore, ribozymes and cofactors collaborate [63,64,65]. This expansion to newly biosynthesized amino acids resembles that envisioned by Wong [66,67]. Wobble arrives late among code assignments because it is mechanistically complex and intrinsically hinders code evolution [54].

Late code evolution slows dramatically for fundamental kinetic reasons [68], thereby hosting a long-lived crescendo [57] of codes with elevated resemblance to the Standard Genetic Code. Mechanistically, continuous escape and diaspora make a near-ideal selection among the crescendo’s random survey of all possible complete codes [58]. Once evolution of ribonucleotide reductase activity [69] makes deoxynucleotide synthesis and the DNA genome possible, a crescendo code becomes the universal SGC in the most successful organism [47] that escapes from its uniquely favorable origin site, flourishing during successful diaspora into more diverse ecological conditions [70]. It is then a short step to the Last Universal Common Ancestor (LUCA) and progressive divergence into Archaea and Bacteria [71].

### 2.11. RNA-Membranes Supply Indispensable Division Functions

Faster, more precise division is an inescapable necessity for evolutionary success among primitive microbes [55]. Figure 4C’s furrow promises [58] just that, ensuring the evolutionary success of the RNA microorganisms that produce it. Thus, Earth’s full biota credibly descend from primordial microbes with Figure 4’s division-promoting RNAs [58].

### 2.12. Modern Membrane RNAs?

The existence of the reactions proposed herein can be investigated with simple experiments, whose methods are already widely employed. A route from existing observations (Figure 2 and Figure 3) to plausible primordial RNA-membranes with protogenomic functions (Figure 4 and Figure 5), or to modern RNA-membranes with established biological roles [3], could be brief indeed.

## Abbreviations

DMPC—1,2-Dimyristoyl-sn-glycero-3-phosphatidylcholine; DOPC—1,2-Dioleoyl-sn-glycero-3-phosphatidylcholine; CHOL—cholesterol; FRET—Förster resonance energy transfer.
